# A glycosylated Phr1 protein is induced by calcium stress and its expression is positively controlled by the calcium/calcineurin signaling transcription factor Crz1 in *Candida albicans*

**DOI:** 10.1186/s12964-023-01224-y

**Published:** 2023-09-18

**Authors:** Linghuo Jiang, Huihui Xu, Yiying Gu, Liudan Wei

**Affiliations:** 1 Laboratory of Yeast Biology and Fermentation Technology, Institute of Biological Sciences and Technology, Guangxi Academy of Sciences, Nanning, 530007 Guangxi China; 2 Department of Food Science, School of Agricultural Engineering and Food Science, Shandong University of Technology, Zibo, 255000 China

**Keywords:** *Candida albicans*, Phr1, Crz1, Glycosylation

## Abstract

As one of the most important human fungal pathogens, *Candida albicans* senses and adapts to host niches with different pH values through the pH-responsive Rim101 pathway. Its transcription factor Rim101 activates the expression of alkaline pH-induced genes including *PHR1* that encodes a glycosylphosphatidylinsitol-anchored β(1,3)-glucanosyltransferase critical for hyphal wall formation. The calcium/calcineurin signaling pathway is mediated by the transcription factor Crz1 in yeasts and other lower eukaryotes. Here we report that deletion of *PHR1* leads to calcium sensitivity of *C. albicans* cells. In addition, expression of Phr1 is induced by calcium stress and under the control of Crz1 in *C. albicans*. EMSA assay demonstrates that Crz1 binds to one CDRE element in the *PHR1* promoter. Alkaline treatment induces two species of glycosylated Phr1 proteins with different degrees of glycosylation, which is independent of Crz1. In contrast, only one species of Phr1 protein with a low degree of glycosylation is induced by calcium stress in a Crz1-dependent fashion. Therefore, we have provided an evidence that regulation of cell wall remodeling is integrated through differential degrees of Phr1 glycosylation by both the pH-regulated Rim101 pathway and the calcium/calcineurin signaling pathway in *C. albicans*.

Video Abstract

**Supplementary Information:**

The online version contains supplementary material available at 10.1186/s12964-023-01224-y.

## Background

As one of the most important human fungal pathogens, *Candida albicans* remains the primary cause of candidiasis, about 60% of the global candidiasis burden [1]. Its cell wall forms the interface with the host, gives the cell shape, and protects cells from stresses and damage. In response to the environmental conditions and host attack, the cell wall allows remodeling and changes in its components, which is composed of the outer mannoprotein coat and the inner polysaccharide-rich layer (chitin and β-glucan cross-linked together). Some cell wall proteins provide anchors for the mannan outer layer of the cell wall, whereas others are cell wall remodeling enzymes responsible for generating essential covalent linkages between cell wall components [2]. The cell wall is essential for the viability of fungal pathogens such as *C. albicans*. In addition, human cells lack a cell wall. Therefore, the fungal cell wall is an attractive target for the development of new antifungal drugs [3, 4].

*C. albicans* can adapt to host niches with different pH values, such as the stomach (pH2.0), the vagina (pH4.5), the kidney, the liver and the duodenum (pH7.0) as well as the blood (pH 7.3) [5, 6]. *C. albicans* cells sense environmental pH changes through the pH-responsive Rim101 pathway that is involved in both tolerance and resistance of antifungal drugs [7, 8]. This pathway is mediated by the transcription factor Rim101, which not only activates the expression of alkaline pH-induced genes such as *PHR1* but also represses the expression of acidic pH-induced genes such as *PHR2* [9, 10]. *C. albicans* Phr1 and Phr2 are members of the GH72 family of glycosylhydrolases, which exist in all fungi and are required for the formation of cell wall [11, 12]. Phr1 and Phr2 are required for proper cross-linking of β-1,3- and β-1,6-glucans in the cell wall of *C. albicans* [13, 14]. Phr1 is required for the virulence of *C. albicans* cells in the mouse model of systemic infection, but not in the rat model of vaginal infection; Contrarily, Phr2 is not required for the virulence of *C. albicans* cells in mouse systemic infection, but is essential in the rat vaginal infection [15]. These data indicate that Phr1 and Phr2 play differential roles in the virulence of *C. albicans* cells. *CRZ1* encodes the transcription factor, a downstream target of the calcium/calcineurin signaling pathway in *C. albicans* [16, 17]. Our recent RNA sequencing work has revealed that Crz1 positively regulates expression of 219 genes, including *PHR1*, in response to calcium stress (Additional file 1: Figure S1; GEO Accession number: GSE123122; [18]). In this study, we have shown that Phr1 is glycosylated and its expression is induced and controlled by Crz1 in response to calcium stress in *C. albicans*.

## Methods

### Strains and media

*C. albicans* strains and plasmids as well as primers used in this study were listed in Table 1 and Additional file 1: Table S1, respectively. LB medium was used for growing bacterial strains. YPD medium and SD medium (0.67% yeast nitrogen base without amino acids, 2% glucose, and auxotrophic amino acids as needed) were used for routine maintaining *C. albicans* strains. Alkaline and acidic treatment was carried out in YPD-150 mM HEPES buffered at pH 8.0 (with NaOH) and 4.0 (with HCl), respectively, as described previously [19]. In brief, cells were grown in YPD medium overnight. Overnight cultures were diluted into YPD-150 mM HEPES buffered at pH 8.0 or 4.0 at a final OD_600nm_ of 0.3, which were allowed to further incubate with shaking for 4 h before cells were collected for protein extraction. It should be noted that the pH-related experiments were conducted steady-states and in adapted cells.

**Table 1:** Strains and plasmids used in this study

Name	Genotype or description	Source
**Strain**		
SN148	Mat*a/α arg4/arg4 leu2/leu2 his1/his1 ura3*::*imm434/ura3*::*imm434*	[20]
HHCA1	SN148 *RPS1/rps1::*CIp10	[18]
HHCA184	SN148* PHR1/PHR1 crz1/crz1*	[18]
HHCA983	SN148* PHR1/PHR1-HA::URA3 crz1/crz1*	This study
HHCA987	SN148* PHR1/PHR1-HA::URA3*	This study
HHCA1033	SN148* PHR1/phr1::ARG4*	This study
HHCA1064	SN148* phr1::HIS1/phr1::ARG4*	This study
HHCA1091	SN148 *phr1:: ARG4/phr1:: HIS1 RPS1/rps1::*CIp10	This study
HHCA1094	SN148 *phr1:: ARG4/phr1:: HIS1 RPS1/rps1::*CIp10-PHR1	This study
HHCA1124	SN148 *PHR1/phr1::ARG4 RPS1/rps1::*CIp10	This study
**Plasmid**		
CIp10	The integration vector (*Amp*^*R*^* URA3*)	[21]
CIp10-PHR1	The full-length *PHR1* gene in CIp10 (*Amp*^*R*^* URA3*)	This study
pFA-HA-URA3	The HA-tag plasmid for HA integration (*Amp*^*R*^* URA3*)	[22]

### Construction of the homozygous mutant for *PHR1*

To construct the homozygous mutant for *PHR1* in the *C. albicans* strain SN148 background, we PCR amplified the *HIS1* and *ARG4* cassettes from the plasmids pFA-HIS1 and pFA-ARG4, respectively, to subsequently replace two alleles of *PHR1* through homologous recombination [23]. Genotypes of the homozygous mutants for *PHR1* were confirmed by PCR (Additional file 1: Figure S2).

### Gene cloning

The full-length gene *PHR1* was PCR amplified with primers PHR1-clone-F/ PHR1-clone-R and cloned between *Kpn*I and *Xho*I sites in the integration vector CIp10 [24], which generated CIp10-PHR1. This recombinant plasmid was confirmed by DNA sequencing, before it was *Stu*I-linearized and integrated into the *RPS1* locus of the homozygous mutant for *PHR1*. The vector CIp10 was *Stu*I-linearized and integrated into the *RPS1* loci of the wild type SN148 as well as its isogenic heterozygous and homozygous mutant for *PHR1*, respectively. Genotypes of these strains were confirmed by PCR with primer pairs CaRPS1-F/URA3-F and CaRPS1-R/CIp10-R [25].

### Chromosomally C-terminal 3xHA tagging of *PHR1*

We PCR amplified the HA-URA3 cassette with primers PHR1-HA-UP/PHR1-HA-DOWN from the plasmid pFA-HA-URA3 [21, 22]. Through homologous recombination approach, we chromosomally integrated the HA tag at the C terminus of Phr1 in the wild type SN148 and the CRISPR homologous mutant for *CaCRZ1* from our previous study [18]. Genotypes of these strains were confirmed by PCR with primer pairs URA3-DF/PHR1-DR and PHR1-DF/HA-DR (Additional file 1: Figures S3).

### Protein extract preparation and deglycosylation reactions

Total protein extracts were prepared and Western blot analysis was carried out as described [26, 27]. In brief, cells were collected by centrifugation and broken by agitation with 5-mm glass beads at 4 °C in protein extraction buffer (50 mM Tris–HCl, pH 7.5/100 mM NaCl/1 mM EDTA/1% NP-40/2 mM PMSF) supplemented with complete protease inhibitor cocktail (Roche Diagnostics, Germany). Total cell lysates were clarified by centrifugation at 10,000 rpm for 10 min at 4 °C. Protein samples were fractionated by SDS-PAGE gels, and transferred onto PVDF membranes. PHR1-HA was detected by Western blot analysis with anti-HA monoclonal antibody. Expression of tubulin was detected with anti-tubulin antibody, which served as an internal expression control.

Protein deglycosylation under both non-denaturing reaction and denaturing reaction conditions was carried out as the manufacturer’s recommendations in the protocol of Protein Deglycosylation Mix II (NEB Biolabs, USA). Briefly, we mixed a total protein sample of 100 μg with 5 μl 10X Deglycosylation Mix Buffer 2, incubated the mixture at 75 °C for 10 min (for denaturing reaction), and cooled it down before the addition of 5 μl Protein Deglycosylation Mix II. For non-denaturing reaction, the mixture was not treated at 75 °C and directly mixed with 5 μl Protein Deglycosylation Mix II. This was followed by an incubation at 25 °C for 30 min and at 37 °C for further 16 h.

### Electrophoretic mobility shift assay (EMSA)

We expressed and purified the recombinant His6-tagged CaCrz1 protein as described previously [18]. The double-stranded probe EMSA_PHR1_F/R was prepared by annealing its two complimentary oligonucleotides (Additional file 1: Table S1), and labeled at the 3′ end with the DIG Gel Shift Kit (Roche) according to the manufacturer’s recommendations.

## Results

### Phr1 is required for the response of *C. albicans* cells to calcium stress

**Fig. 1:**
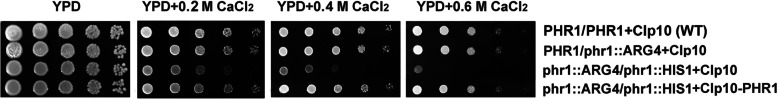
Phenotypes of *Candida albicans* cells lacking a functional *PHR1* gene. The wild type SN148 (WT; HHCA1), its isogenic heterozygous (*PHR1/phr1::ARG4*; HHCA1124) and homozygous (*phr1::ARG4/phr1::HIS1*; HHCA1091) mutants for *PHR1* as well as the complemented strain (*phr1::ARG4/phr1::HIS1* + CIp10-PHR1; HHCA1094) were grown overnight at 30 °C in liquid SD-URA medium, and overnight cultures were serially diluted and spotted onto YPD plates with or without CaCl_2_. Plates were incubated for 2–3 days before photos were taken

In order to examine if Phr1 is involved in the response of *C. albicans* cells to calcium stress, we generated the homozygous mutant for *PHR1* by replacing the two alleles of *PHR1* with the *ARG4* cassette and the *HIS1* cassette, respectively. Consistent with previous studies [28, 29], the homozygous mutant for *PHR1* was sensitive to alkaline treatment but not to an acidic treatment (Additional file 1: Figures S4). As compared to the wild type and the heterozygous mutant for *PHR1*, the homozygous mutant for *PHR1* was sensitive to 0.4 M CaCl_2_ and 0.6 M CaCl_2_, but not to 0.2 M CaCl_2_, and introduction of *PHR1* back to its homozygous mutant reversed its calcium-sensitive phenotype (Fig. 1; Additional file 1: Figure S5). As compared to the wild type, we did not observe a change in the sensitivity of the homozygous mutant for *PHR1* to 0.4 M MgCl_2_, 0.4 M MnCl_2_, 0.4 M LiCl, 0.4 M KCl, and 0.4 M NaCl (Data now shown). Taken together, these data indicate that Phr1 is required for the response of *C. albicans* cells to calcium stress.

### Calcium-induced expression of Phr1 is, but alkaline-induced expression of Phr1 is not, dependent of the transcription factor CaCrz1

*PHR1* is known to be one of the downstream targets of the Rim101 signaling in *C. albicans* [9, 10]. Expression level of *PHR1* is barely detectable at pH4.0 and significantly induced at pH8.0, and this alkaline-induction is controlled by Rim101 [30]. In our previous study [18], we have observed that transcripts of *PHR1* is induced by calcium stress, which could be abolished in the absence of Crz1 (Additional file 1: Figure S1). To examine the protein expression of Phr1 in response to calcium stress and pH conditions, we chromosomally 3xHA tagged the C-terminus of one *PHR1* allele in both the wild type SN148 and its isogenic homozygous mutant for *CaCRZ1* (Additional file 1: Figure S3).

**Fig. 2:**
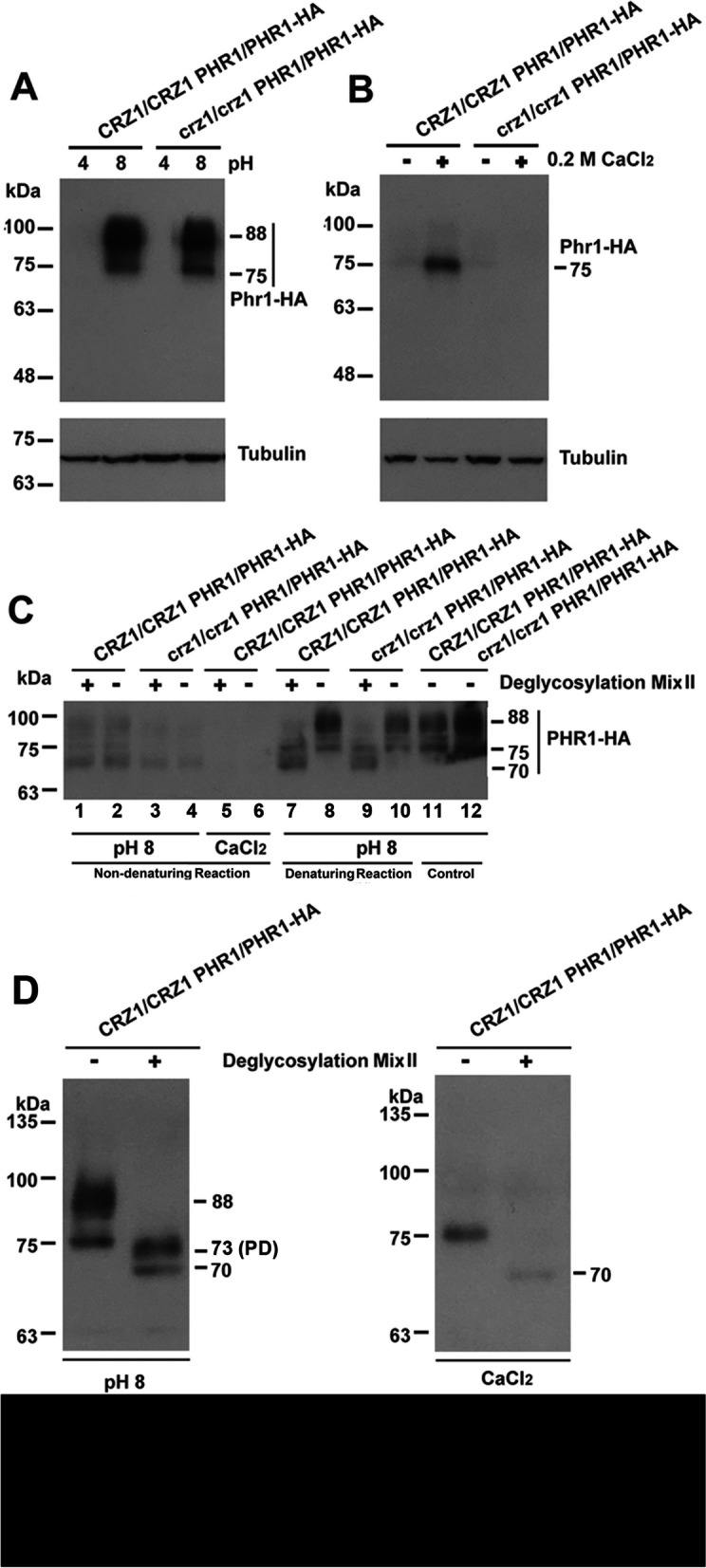
Expression and deglycosylation of Phr1-HA proteins induced by alkaline treatment and calcium stress. Log-phase growing *CRZ1/CRZ1 PHR1/PHR1-HA* and *crz1/crz1 PHR1/PHR1-HA* cells were treated in YPD-150 mM HEPES buffered at pH 4.0 or 8.0 (**A**) or in YPD containing 0.2 M CaCl_2_ (**B**) for one hour, before total protein samples were extracted from these cells and subjected for Western blot analysis with anti-HA or anti-tubulin (as internal control) antibodies. Two times of western blots were carried out. Total protein samples were also subjected for deglycosylation analysis under both non-denaturing and denaturing reaction conditions (**C**) and only under denaturing reaction condition (**D**). PD, partial deglycosylation

In both the wild type and the *crz1/crz1* strains, two species of Phr1-HA proteins with approximately sizes of 88 kDa and 75 kDa were induced by alkaline treatment, but not by acidic treatment, with the 88-kDa protein being more abundant (Fig. 2A). Both Phr1-HA bands were bigger than its expected size of 63 kDa, suggesting posttranslational modifications for Phr1-HA proteins. This is consistent with a previous observation on the Phr1 protein tagged with GFP, showing a posttranslational modification with a possible glycosylation degree similar to the 88-kDa Phr1-HA band [19]. In addition, a mass spectrometric quantification approach detected the presence of Phr1 in the cell wall of cells growing under only alkaline but not acidic conditions [31]. Our result indicates that alkaline-induced expression of Phr1 is independent of CaCrz1. In contrast, in response to 0.2 M CaCl_2_, a Phr1-HA protein of about 75 kDa was also induced in the wild type strain, but not in the *crz1/crz1* strain (Fig. 2B). This suggests that the calcium-induced expression of the 75-kDa Phr1-HA protein is dependent of CaCrz1.

### Alkaline- and calcium-induced Phr1 proteins are differentially glycosylated

Phr1 is a regulator of cell wall remodeling. We next tested if Phr1 proteins were glycosylated. When protein samples were not treated at 75 °C for 10 min (non-denaturing condition) before they were subjected for deglycosylation digestion, we observed that the alkaline-induced 88-kDa Phr1-HA product from both the wild type and the *crz1/crz1* strains was largely deglycosylated to be a band of about 70 kDa (Lanes 1–4; Fig. 2C). When protein samples were pretreated at 75 °C for 10 min (denaturing condition), the 88-kDa Phr1-HA protein was deglycosylated to generate a smaller protein of about 73 kDa; whereas the 75-kDa Phr1-HA protein was deglycosylated to be a 70-kDa protein (Lanes 7–10; Fig. 2C and left panel; D). The 73-kDa band derived from the deglycosylation of the alkaline-induced 88-kDa Phr1-HA protein under denaturing condition seems to be not as sharp as the 70-kDa band (left panel; Fig. 2D), which indicates the 73-kDa product might be a partially deglycosylated form of the 88-kDa protein.

We did not observe any band from the deglycosylation reaction of two CaCl_2_-induced Phr1-HA samples under non-denaturing condition (lanes 5 and 6; Fig. 2C). This should be caused by the lower expression level of the 75-kDa Phr1-HA product induced by calcium stress and a possible protein degradation under non-denaturing condition, because a significant general protein degradation occurred during deglycosylation digestion if protein samples were not pretreated at 75 °C for 10 min (compare signal intensities between lanes 1–4 and lanes 7–10; Fig. 2C). After we increased the total protein amount to 200 μg in the deglycosylation reaction, a deglycosylated 70-kDa product was also observed in the calcium-induced Phr1-HA sample under denaturing condition (right panel; Fig. 2D). Taken together, these results suggest that both the 88-kDa and the 75-kDa Phr1-HA proteins are glycosylated.

### CaCrz1 binds to the *PHR1* promoter *in vitro*

**Fig. 3:**
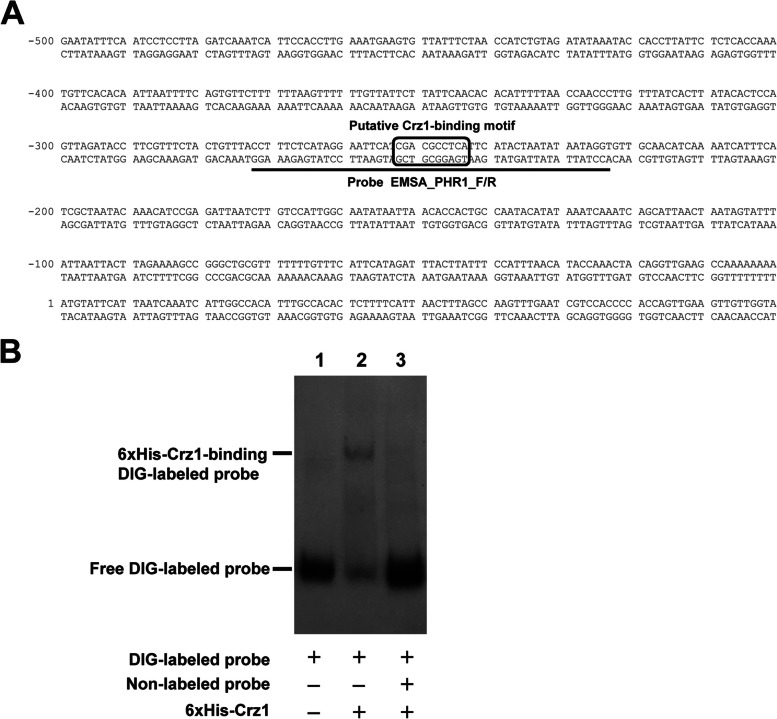
CaCrz1 binds in vitro to the *PHR1* promoter. **A** Location of the predicated CaCrz1-binding motif (boxed) in the promoter of *PHR1*. Location of the double-stranded probe EMSA_PHR1_F/R sequence is indicated underlined with a solid line. **B** DIG-labelled Probe EMSA_PHR1_F/R was added into samples in Lanes 1–3. The unlabelled Probe EMSA_PHR1_F/R was added into the sample in Lane 3. Purified 6xHis-Crz1 protein of 1 μg was added into samples in Lanes 2 and 3, respectively. Two EMSA replicates were performed

We identified one potential CaCrz1-binding site from the complementary sequence of the 5’ CGACGCCTCA 3’, which locates at -244 to -253 from the ORF start, in the promoter of *PHR1* according to the consensus motif in promoters of CaCrz1 target genes identified previously (Fig. 3A; [18]). To determine if CaCrz1 binds to the CDRE motif in the *PHR1* promoter, we carried out EMSA experiment. The EMSA assay demonstrated that the recombinant 6xHis-Crz1 protein bound to the DIG-labeled probe (Lane 2 in Fig. 3B). The binding of 6xHis-Crz1 to the probe was abolished by its specific competitor, the unlabeled probe (Lane 3 in Fig. 3B). Taken together, these data indicate that CaCrz1 can bind in vitro to the CDRE motif in the *PHR1* promoter.

## Discussion

It is known that in response to alkaline treatment, Rim101 directly binds to the promoter of the pH-responsive gene *PHR1* to induce its transcriptional expression in *C. albicans* [10]. Here, we have demonstrated that *C. albicans* cells lacking *PHR1* are sensitive to calcium stress. Furthermore, expression of Phr1 is also induced by calcium stress, which is controlled by the transcription factor CaCrz1 likely through its CDRE motif [5’ C_253_GACGCCTCA 3’] in the promoter of *PHR1*. In *Saccharomyces cerevisiae*, exposure of cells to an alkaline environment produces a robust transcriptional response involving hundreds of genes, part of which is triggered by an immediate burst of calcium and recruitment of Crz1 to the promoters of more than 100 genes [32, 33]. Immediate calcium influx has also been observed in the response of *C. albicans* cells to alkaline treatment, and expression of alkaline-induced *PHO85* has been shown to be positively controlled by the calcium signaling transcription factor Crz1 [34]. In this study, we have shown that the Phr1 expression/glycosylation in alkaline pH is not dependent on CaCrz1, although alkaline pH can trigger calcium influx to the cell. This could be due to the possibility that Rim101-binding might somehow hinder the binding of CaCrz1 to its CDRE motif in the promoter of *PHR1*. In *C. albicans*, the Rim101 pathway acts in parallel to Crz1, via calcineurin, to adapt to alkaline pH, and acts in parallel to the Crz1 homologue Crz2, independent of calcineurin, to adapt to lithium stress and to repress filamentation at acidic pH [35]. Our study has provided an additional example that expression of an alkaline-induced cell wall-related gene *PHR1* is also positively controlled by Crz1, the transcription factor of the calcium/calcineurin signaling pathway in *C. albicans*.

In addition, we have demonstrated that Phr1 proteins induced by both alkaline and calcium stresses are glycosylated. Two species of Phr1 proteins are observed to be induced by alkaline treatment. The smaller glycosylated species induced by alkaline treatment shows a similar size to the only one induced by calcium stress, but they might be different glycosylation species because they are controlled by Rim101 and Crz1, respectively (Fig. 2A). To our knowledge, this is the first report to demonstrate that Phr1 proteins are glycosylated in *C. albicans*. Our results are consistent with the bioinformatic analysis of Phr1 amino acid sequence by Psort II (https://psort.hgc.jp/form2.html), which predicts that Phr1 has a cleavable N-terminal signal peptide (M1 to A20) and is likely to be GPI anchored. In addition, there is a serine-rich region in the C-terminal region (486S-517S) of Phr1 protein (http://www.candidagenome.org/cgi-bin/locus.pl?locus=phr1&organism = C_albicans_ SC5314). These three characteristics meet all criteria for being GPI-anchored plasma membrane mannoproteins in *S. cerevisiae* [36, 37]. In this study, we have shown that the deglycosylated band of Phr1-HA is 70 kDa (Fig. 2D), which is still bigger than its expected size of about 63 kDa. This difference might be caused by another kind of posttranslational modification such as phosphorylation, which needs to be further addressed in the future study.

### Supplementary Information

**Additional file 1:** **Figure S1. **Transcript levels of *PHR1*genein the wild type SN148 and its isogenic mutant *crz1/crz1*cells growing in log phase in the presence or absence of0.2M CaCl2for 2 hours. **Figure S2. **Knockoutstrategy of two alleles of *PHR1*and PCR confirmation of genotypes. **Figure S3. **Chromosomally C-terminal 3xHA tagging of *PHR1. ***Figure S4. **Deletion of *PHR1*leads to sensitivity of *C. albicans*cells toalkaline stress. **Figure S5. **Cation sensitivityof*Candida albicans*cells lacking a functional *PHR1*gene. **Table S1. **Primers used in this study.

## Data Availability

All data generated or analyzed during this study are included in this published article.

## Abbreviations

Crz1: Calcineurin-responsive zinc finger 1
PCR: Polymerase chain reaction
YPD: Yeast peptone dextron
