# Supplementary material for: A glycosylated Phr1 protein is induced by calcium stress and its expression is positively controlled by the calcium/calcineurin signaling transcription factor Crz1 in Candida albicans
Source: Cell Commun Signal. 2023 Sep 18;21:237. doi: 10.1186/s12964-023-01224-y (PMC10506259; doi:10.1186/s12964-023-01224-y)
Supplement: Supplementary file 2 — Additional file 1: Figure S1. Transcript levels of PHR1genein the wild type SN148 and its isogenic mutant crz1/crz1cells growing in log phase in the presence or absence of0.2M CaCl2for 2 hours. Figure S2. Knockoutstrategy of two alleles of PHR1and PCR confirmation of genotypes. Figure S3. Chromosomally C-terminal 3xHA tagging of PHR1. Figure S4. Deletion of PHR1leads to sensitivity of C. albicanscells toalkaline stress. Figure S5. Cation sensitivityofCandida albicanscells lacking a functional PHR1gene. Table S1. Primers used in this study. [file 12964_2023_1224_MOESM1_ESM.zip › Additional file 1 Figure S2.pdf]

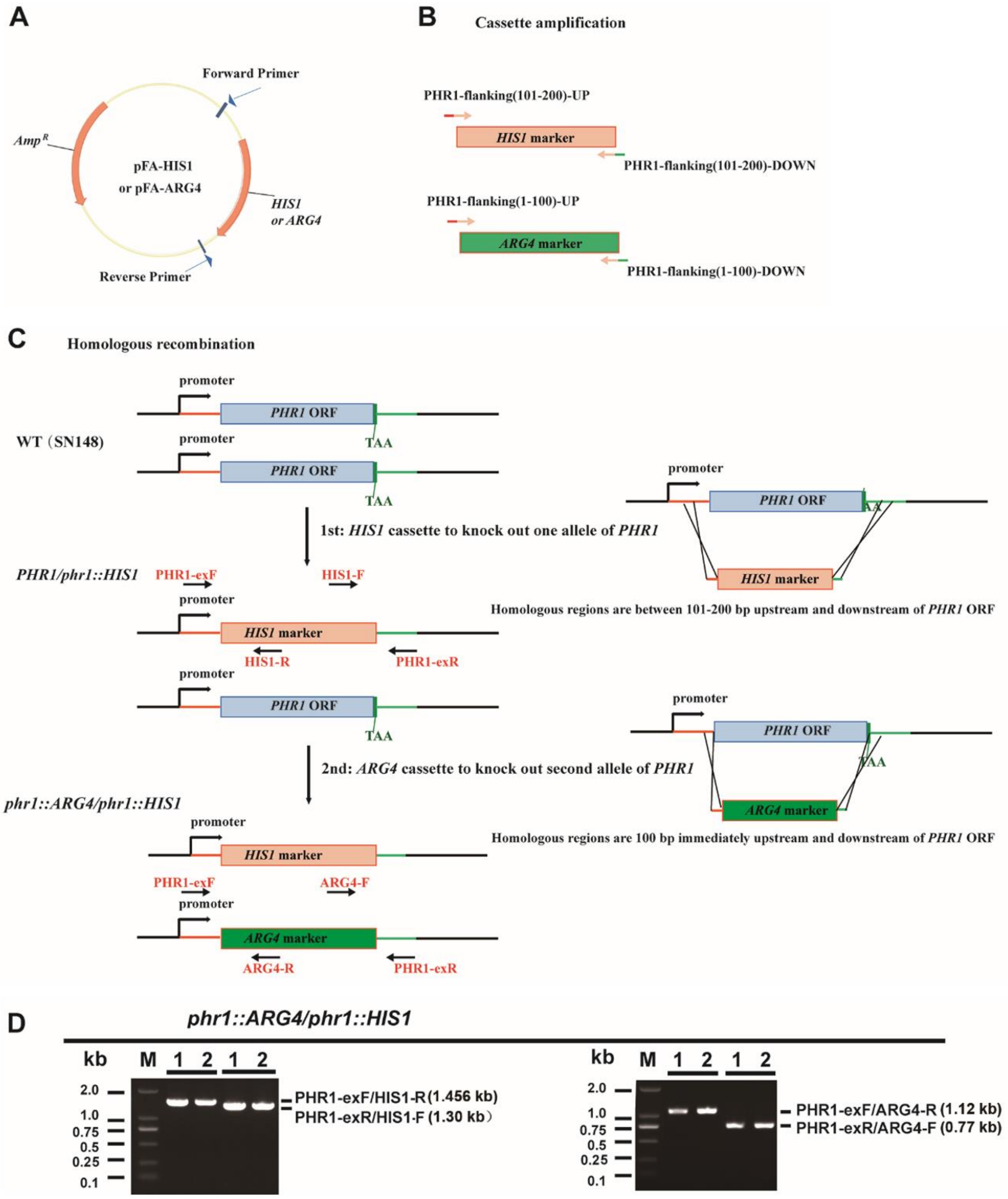

Figure S2. Knockout strategy of two alleles of *PHR1* and PCR confirmation of genotypes. A, Restriction maps of pFA-ARG4 and pFA-HIS1 plasmids. **B,** PCR amplification of *ARG4* and *HIS1* cassettes. **C,** knockout steps. **D,** PCR confirmation of genotypes for two independent homozygous mutants for *PHR1* (No. 1 and No.2). Expected PCR products were amplified with four pairs of primers indicated on the right of gels.
