# Supplementary material for: A glycosylated Phr1 protein is induced by calcium stress and its expression is positively controlled by the calcium/calcineurin signaling transcription factor Crz1 in Candida albicans
Source: Cell Commun Signal. 2023 Sep 18;21:237. doi: 10.1186/s12964-023-01224-y (PMC10506259; doi:10.1186/s12964-023-01224-y)
Supplement: Supplementary file 2 — Additional file 1: Figure S1. Transcript levels of PHR1genein the wild type SN148 and its isogenic mutant crz1/crz1cells growing in log phase in the presence or absence of0.2M CaCl2for 2 hours. Figure S2. Knockoutstrategy of two alleles of PHR1and PCR confirmation of genotypes. Figure S3. Chromosomally C-terminal 3xHA tagging of PHR1. Figure S4. Deletion of PHR1leads to sensitivity of C. albicanscells toalkaline stress. Figure S5. Cation sensitivityofCandida albicanscells lacking a functional PHR1gene. Table S1. Primers used in this study. [file 12964_2023_1224_MOESM1_ESM.zip › Additional file 1 Figure S3.pdf]

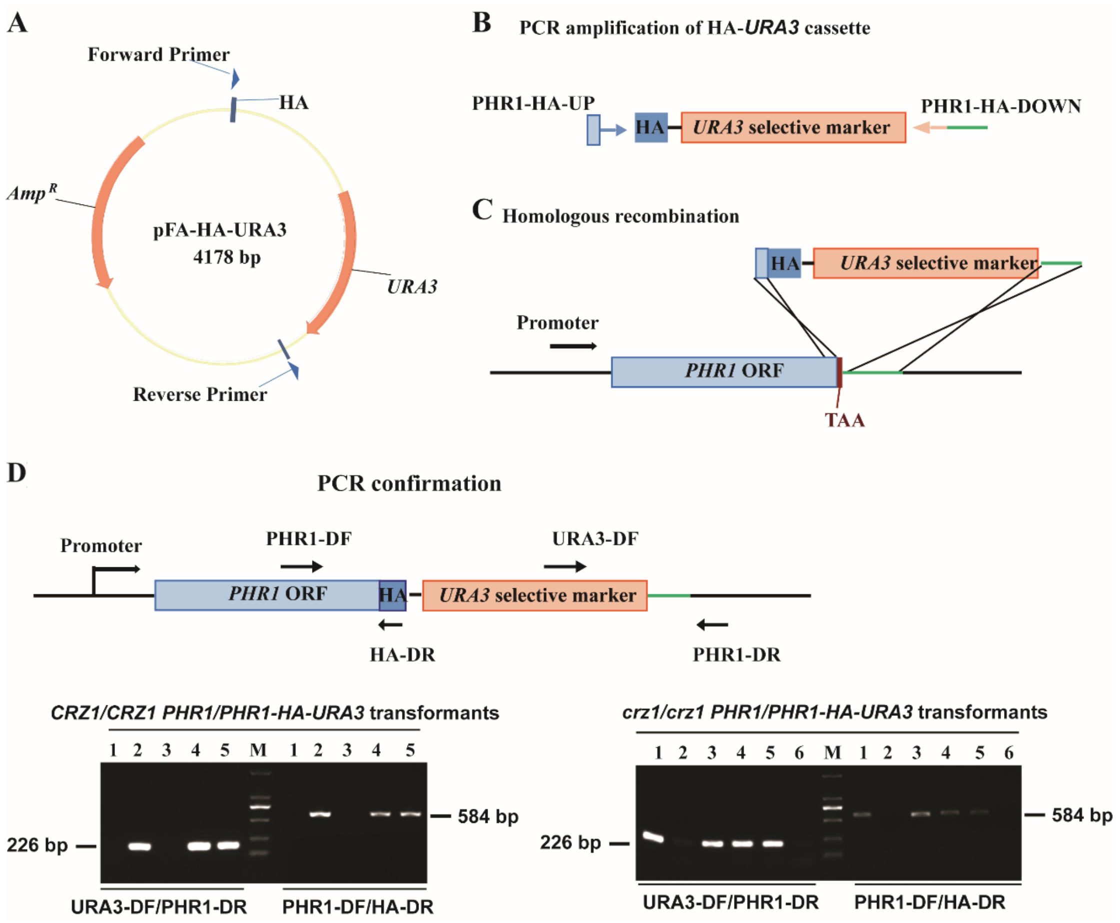

Figure S3. Chromosomally C-terminal 3xHA tagging of *PHR1*

A, restriction map of the pFA-HA-URA3 plasmid. **B,** PCR amplification of the HA-URA3 cassette from pFA-HA-URA3 with primers PHR1-HA-UP and PHR1-HA-DOWN. **C,** the strategy for chromosomally tagging HA to the C-terminus of CaPhr1. **D**, PCR confirmation of correct integration of the GFP-URA3 cassette in the genomes of the wild-type SN148 (*CRZ1/CRZ1 PHR1/PHR1*) (left panel) and its isogenic CRISPR inactivation mutant for *CaCRZ1* (*crz1/crz1 PHR1/PHR1*) (Right panel). PCR products of 266-bp and 584-bp were amplified with primer pairs URA3-DF/PHR1-DR and PHR1-DF/HA-DR, respectively, from genomic DNA sample of three independent transformants No. 2, No. 4 and No.5, but not from No. 1 and No.3, of the wild-type SN148. Similarly, PCR products of 266-bp and 584-bp were amplified with primer pairs URA3-DF/PHR1-DR and PHR1-DF/HA-DR, respectively, from genomic DNA sample of four independent transformants No. 1, No. 3, No. 4 and No.5, but not from No. 2 and No.6, of the mutant for *CaCRZ1*.
