# Supplementary material for: A glycosylated Phr1 protein is induced by calcium stress and its expression is positively controlled by the calcium/calcineurin signaling transcription factor Crz1 in Candida albicans
Source: Cell Commun Signal. 2023 Sep 18;21:237. doi: 10.1186/s12964-023-01224-y (PMC10506259; doi:10.1186/s12964-023-01224-y)
Supplement: Supplementary file 2 — Additional file 1: Figure S1. Transcript levels of PHR1genein the wild type SN148 and its isogenic mutant crz1/crz1cells growing in log phase in the presence or absence of0.2M CaCl2for 2 hours. Figure S2. Knockoutstrategy of two alleles of PHR1and PCR confirmation of genotypes. Figure S3. Chromosomally C-terminal 3xHA tagging of PHR1. Figure S4. Deletion of PHR1leads to sensitivity of C. albicanscells toalkaline stress. Figure S5. Cation sensitivityofCandida albicanscells lacking a functional PHR1gene. Table S1. Primers used in this study. [file 12964_2023_1224_MOESM1_ESM.zip › Additional file 1 Figure S4.pdf]

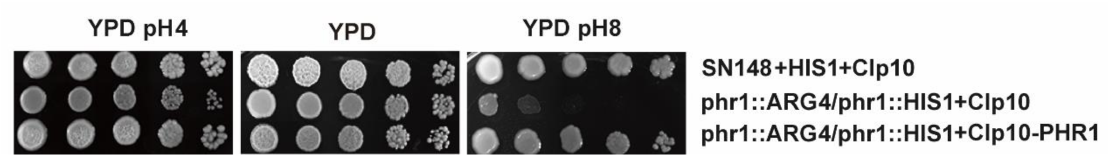

Figure S4. Deletion of *PHR1* leads to sensitivity of *C. albicans* cells to alkaline stress. The wild type SN148, the homozygous mutant for *PHR1* and the complemented strain were grown overnight in liquid SD-URA medium, diluted serially by 10 times and spotted onto the regular YPD plate (middle panel) as well as YPD plates adjusted to pH 4.0 (left panel) and pH8.0 (right panel). Plates were incubated at 30°C for 2-3 days before photos were taken.
