# Supplementary material for: A glycosylated Phr1 protein is induced by calcium stress and its expression is positively controlled by the calcium/calcineurin signaling transcription factor Crz1 in Candida albicans
Source: Cell Commun Signal. 2023 Sep 18;21:237. doi: 10.1186/s12964-023-01224-y (PMC10506259; doi:10.1186/s12964-023-01224-y)
Supplement: Supplementary file 2 — Additional file 1: Figure S1. Transcript levels of PHR1genein the wild type SN148 and its isogenic mutant crz1/crz1cells growing in log phase in the presence or absence of0.2M CaCl2for 2 hours. Figure S2. Knockoutstrategy of two alleles of PHR1and PCR confirmation of genotypes. Figure S3. Chromosomally C-terminal 3xHA tagging of PHR1. Figure S4. Deletion of PHR1leads to sensitivity of C. albicanscells toalkaline stress. Figure S5. Cation sensitivityofCandida albicanscells lacking a functional PHR1gene. Table S1. Primers used in this study. [file 12964_2023_1224_MOESM1_ESM.zip › Additional file 1 Figure S5.pdf]

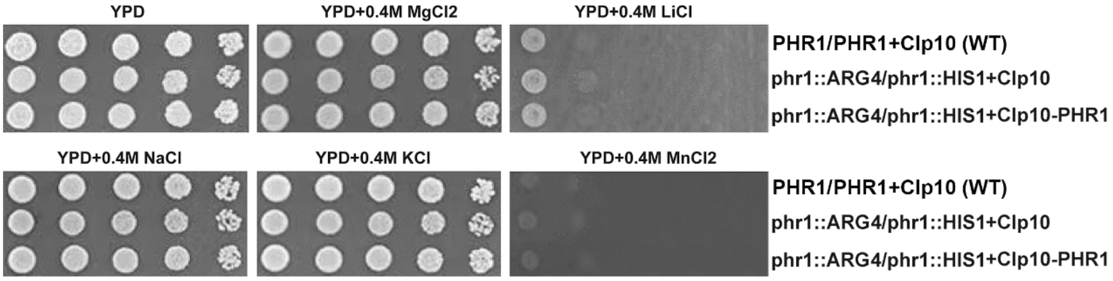

Additional file 1 Figure S5. Cation sensitivity of *Candida albicans* cells lacking a functional *PHR1* gene. The wild type SN148 (WT; HHCA1), its isogenic homozygous (*phr1::ARG4/phr1::HIS1*; HHCA1091) mutant for *PHR1* as well as the complemented strain (*phr1::ARG4/phr1::HIS1*+CIp10-PHR1; HHCA1094) were grown overnight at 30°C in liquid SD-URA medium, and overnight cultures were serially diluted and spotted onto YPD plates with or without indicated cations. Plates were incubated for 2-3 days before photos were taken.
