# Supplementary material for: A glycosylated Phr1 protein is induced by calcium stress and its expression is positively controlled by the calcium/calcineurin signaling transcription factor Crz1 in Candida albicans
Source: Cell Commun Signal. 2023 Sep 18;21:237. doi: 10.1186/s12964-023-01224-y (PMC10506259; doi:10.1186/s12964-023-01224-y)
Supplement: Supplementary file 2 — Additional file 1: Figure S1. Transcript levels of PHR1genein the wild type SN148 and its isogenic mutant crz1/crz1cells growing in log phase in the presence or absence of0.2M CaCl2for 2 hours. Figure S2. Knockoutstrategy of two alleles of PHR1and PCR confirmation of genotypes. Figure S3. Chromosomally C-terminal 3xHA tagging of PHR1. Figure S4. Deletion of PHR1leads to sensitivity of C. albicanscells toalkaline stress. Figure S5. Cation sensitivityofCandida albicanscells lacking a functional PHR1gene. Table S1. Primers used in this study. [file 12964_2023_1224_MOESM1_ESM.zip › Additional file 1 Table S1.pdf]

Table S1 Primer s used in this study

Primer name	Sequence (5'-3')
PHR1-flanking(1-100)-UP	TAATTACTTAGAAAAGCCGGGCTGCGTTTTTTTGTTTCATTCATAGATTTACTTATTTCCATTTAACATACCAAACTACAGGTTGAAGCCAAAAAAAAgaagcttcgtacgctgcaggtc
PHR1-flanking(1-100)-DOWN	ATTTCAAATTGGAACTTTTAAATAATGAACTCTTATTAAACTATATATATTCGTCCTATACAACAGAACCCTTCATATCGGTTAGTCTTCATTGATtctgatatcatcgatgaattcgag
PHR1-flanking(101-200)-UP	GCTAATACAAACATCCGAGATTAATCTTGTCCATTGGCAATATAATTAACACCACTGCCAATACATATAAATCAAATCAGCATTAACTAATAGTATTTgaagcttcgtacgctgcaggtc
PHR1-flanking(101-200)- DOWN	GATCACCAATTGAGAAACTTAGGGATGGTATAAATAAACTAGGACAATTTGTAATGCATGTACTACAATAGATAGAGAATAGATATGTGTACACAAtctgatatcatcgatgaattcgag
PHR1-exF	CAACACACATTTTTAACCAACCC
PHR1-exR	CGAACTGGTTCAGATGAAACAC
ARG4-F	GGATATGTTGGCTACTGATTTAGC
ARG4-R	GTATGAATATCCTCATCACCAGCC
HIS1-F	TTTAGTCAATCATTTACCAGACCG
HIS1-R	GGTTGCACCAGCTTTCTTC
PHR1-clone-F	agggaacaaaagctgggtaccCGTCCATGACATTTACTCTACCG (*Kpn*I site underlined)
PHR1-clone-R	atcgataccgtcgacctcgagAAAGGGCATCAGTGATGGATTC (*Xho*I site underlined)
EMSA_PHR1_F	CCTTTCTCAT AGGAATTCAT CGACGCCTCA TTCATACTAA TAT AATAGGT
EMSA_PHR1_R	GGAAAGAGTA TCCTTAAGTA GCTGCGGAGT AAGTATGATT ATATTATCCA
PHR1-HA-UP	CTCAACAAATGTCTATGGTCAAATTGGTTTCAATTATTACTATTGTTACTGCATTTGTTGGTGGTATGTCCGTTGTTTTT**CCCGGGTACCCATACGATGT**
PHR1-HA-Down	TCAGCTAGATCAAAGTGGATTAAGCAAAAAAGGGCATCAGTGATGGATTCAAAAGCATTAAACGAAATTTTCATGATCACC**CGCATAGGCCACTAGTGGA**
PHR1-DF	CCTCTGCATCTGCTTCTGG
PHR1-DR	CGAACTGGTTCAGATGAAACAC
HA-DR	GGATATCCTGCATAGTCC
URA3-DF	CCAACGTGATATGGGTGGC
PHR1-exF	CCTCTGCATCTGCTTCTGG
PHR1-exR	CGAACTGGTTCAGATGAAACAC
CaRPS1-F	GTCGTCTGAATGTCGCAAC
CaRPS1-R	GCCATAAATATGCCGATTCTC
URA3-F(Common primer)	GGTGACACTATAGAACGCG
CIp10-R	GACCTACACCGAACTGAGATAC
